# Single‐Voxel Proton Magnetic Resonance Spectroscopy Findings at 3 Tesla in a Dog With Gliomatosis Cerebri

**DOI:** 10.1111/jvim.70210

**Published:** 2025-09-25

**Authors:** Péter Sebestyén, Chris Staudinger, Robert Herzig, Anna Oevermann, Lorenzo Golini, Niklaus Zölch, Katrin Beckmann

**Affiliations:** ^1^ Section of Neurology, Department of Small Animals, Vetsuisse Faculty University of Zurich Zurich Switzerland; ^2^ Clinic for Diagnostic Imaging, Department of Clinical Diagnostics and Services, Vetsuisse Faculty University of Zurich Zurich Switzerland; ^3^ Division of Neurological Sciences, Vetsuisse Faculty University of Bern Bern Switzerland; ^4^ Department of Forensic Medicine and Imaging, Institute of Forensic Medicine University of Zurich Zurich Switzerland; ^5^ Department of Psychiatry, Psychotherapy and Psychosomatics, Psychiatric Hospital University of Zurich Zurich Switzerland

**Keywords:** 1H‐MRS, bilateral symmetric lesions, intracranial neoplasia, myo‐inositol, NAA, neuroimaging

## Abstract

Gliomatosis cerebri (GC) represents an antemortem diagnostic challenge in the absence of histopathology. Proton magnetic resonance spectroscopy (1H‐MRS) features of the disease in humans include elevated myo‐inositol (mI)‐to‐creatine and decreased *N‐*acetyl‐aspartate (NAA)‐to‐creatine ratios. Brain 1H‐MRS findings at 3 Tesla (3 T) field strength in dogs with GC have not yet been described. A 12‐year‐old West Highland White Terrier was presented with a progressive history of multifocal encephalopathy. A 3 T MRI revealed a diffuse, bilateral, ill‐defined, intra‐axial white matter lesion that was T2W and FLAIR hyperintense, T1W iso‐ to hypointense, showed no contrast enhancement, and was associated with moderate mass effect. 1H‐MRS with voxel positioning at the left parietal area showed highly elevated mI and decreased NAA levels compared to healthy control dogs measured using the same protocol in the thalamus. GC was confirmed by stereotactic brain biopsy. Comparable 1H‐MRS changes to those reported in humans were identified in a dog with GC.

**Abbreviations**
1H‐MRS: proton magnetic resonance spectroscopy
3 T: 3 Tesla
ADC: apparent diffusion coefficient
CSF: cerebrospinal fluid
DWI: diffusion weighted imaging
FLAIR: fluid‐attenuated inversion recovery
GC: gliomatosis cerebri
Glu: glucose
Glx: glutamate‐glutamine complex
mI: myo‐inositol
MRI: magnetic resonance imaging
MUO: meningoencephalitis of unknown origin
NAA: *N*‐acetyl‐aspartate
SWI: susceptibility weighted imaging
T1W: T1 weighted
T2W: T2 weighted
tCho: total choline
tCr: total creatine
TE: echo time
tNAA: *N*‐acetyl‐aspartate containing compounds

## 1 Introduction

Gliomatosis cerebri (GC), according to the latest World Health Organization (WHO) classification, is no longer considered a distinct neoplasia with a specific cellular phenotype, but rather a growth pattern of a glial cell tumor characterized by a diffuse infiltration of more than two cerebral lobes with or without involvement of deep gray matter structures, brainstem, cerebellum, or spinal cord [1, 2]. Most GCs are of astrocytic origin, although both oligodendroglial and mixed phenotypes have been described [2, 3]. This tumor carries a grave prognosis and is rarely reported in people and domestic animals [2, 4, 5, 6, 7, 8, 9, 10, 11, 12, 13, 14]. The clinical signs of GC are variable and depend mostly on the location and extent of neoplastic infiltration [11, 14]. Due to the heterogeneous nature of this neoplasm, in vivo diagnosis without histopathological examination is challenging and primarily relies on diagnostic imaging [14, 15].

Conventional magnetic resonance imaging (MRI) findings which could indicate GC in dogs are ill‐defined, T1‐weighted (T1W) hypo‐ or isointense and T2‐weighted (T2W) and fluid‐attenuated inversion recovery (FLAIR) hyperintense lesions with minimal or no contrast enhancement, affecting white matter or both white and gray matter in at least three contiguous brain regions, with occasional involvement of the thalamus and caudal fossa structures [14]. However, these findings are nonspecific and overlap with MRI features of inflammatory, metabolic, or toxic brain diseases [14, 15, 16].

Proton magnetic resonance spectroscopy (1H‐MRS) is a non‐invasive diagnostic imaging method that complements conventional MRI and is used to measure tissue metabolite concentration or their ratios to each other, improving diagnostic accuracy for intracranial diseases [17, 18, 19, 20]. Commonly measured metabolites with 1H‐MRS include *N*‐acetyl‐aspartate (NAA), choline, creatine, and myo‐inositol (mI) [21]. Due to the limited spectral resolution in vivo, it is generally not possible to unambiguously distinguish between metabolites with highly similar chemical structures. Consequently, the reported values typically represent composite signals: total NAA (tNAA), comprising NAA and *N*‐acetyl‐aspartyl‐glutamate; total creatine (tCr), encompassing both creatine and phosphocreatine; and total choline (tCho), which includes all choline‐containing compounds [17, 22, 23]. To date, 1H‐MRS characterization of GC in dogs is limited to a single case report measured at 1.5 Tesla with a long echo time (TE) of 144 ms and poor spectral quality. This report found decreased levels of tNAA relative to both tCho and tCr [13]. In humans, another important 1H‐MRS feature of GC is a marked elevation in mI levels [24, 25, 26]. Myo‐inositol is a cyclic sugar alcohol believed to be a marker of glial proliferation. It is readily detected in short TE 1H‐MRS spectra of the healthy dog and human brain but overlaps with glycine [27]. To the authors' knowledge, it has not yet been reported in association with GC in dogs.

In this case report, we describe the conventional MRI and 1H‐MRS findings at 3 Tesla (3 T) of a dog with GC. We also compare the 1H‐MRS values to published reference ranges from healthy control dogs and from dogs with encephalopathies exhibiting similar conventional MRI findings to those observed in this case with GC, as well as to values reported in humans with GC.

## 2 Case Description

A 12‐year‐old female neutered West Highland White Terrier was referred to the neurology service of the small animal clinic of the University of Zurich with anorexia, behavior changes, and progressive neurological deficits that had been present for 3 weeks. Three days before presentation, hematology and comprehensive biochemistry at the referring veterinarian were within normal limits, and the dog was pretreated with fluids, maropitant (1 mg/kg q24h; Cerenia, Zoetis Schweiz GmbH, Delémont, Switzerland) and metronidazole (15 mg/kg q8h; Metronidazole, B. Braun Melsungen AG, Melsungen, Germany) administered intravenously.

At presentation, clinical examination and the blood pressure were within normal limits. The neurological examination revealed disoriented mental status, bilateral reduced menace response with normal pupillary light reflexes, generalized proprioceptive ataxia with delayed paw positioning in all limbs, and normal spinal reflexes. Neuroanatomical localization was consistent with multifocal brain lesion (forebrain and brainstem). Based on the signalment and neurological deficits, a structural brain disease such as a meningoencephalitis of infectious or noninfectious origin or an infiltrative central nervous system neoplasia was suspected.

Thoracic radiographs revealed no evidence of metastatic disease. Brain MRI was performed under general anesthesia using a 3 T scanner (Philips Ingenia, Philips AG, Zurich, Switzerland). The imaging protocol consisted of T2W, FLAIR, susceptibility weighted imaging (SWI), and pre‐ and postcontrast 3D T1W sequences in transverse, sagittal, and dorsal planes. In addition, transverse diffusion weighted imaging (DWI) was performed, and the apparent diffusion coefficient (ADC) map was calculated. Postcontrast images were obtained after administration of a gadolinium‐based contrast agent (0.1 mmol/kg; Gadoterate meglumine, Dotarem, Guerbet GmbH, Zurich, Switzerland) (Table S1).

**FIGURE 1:**
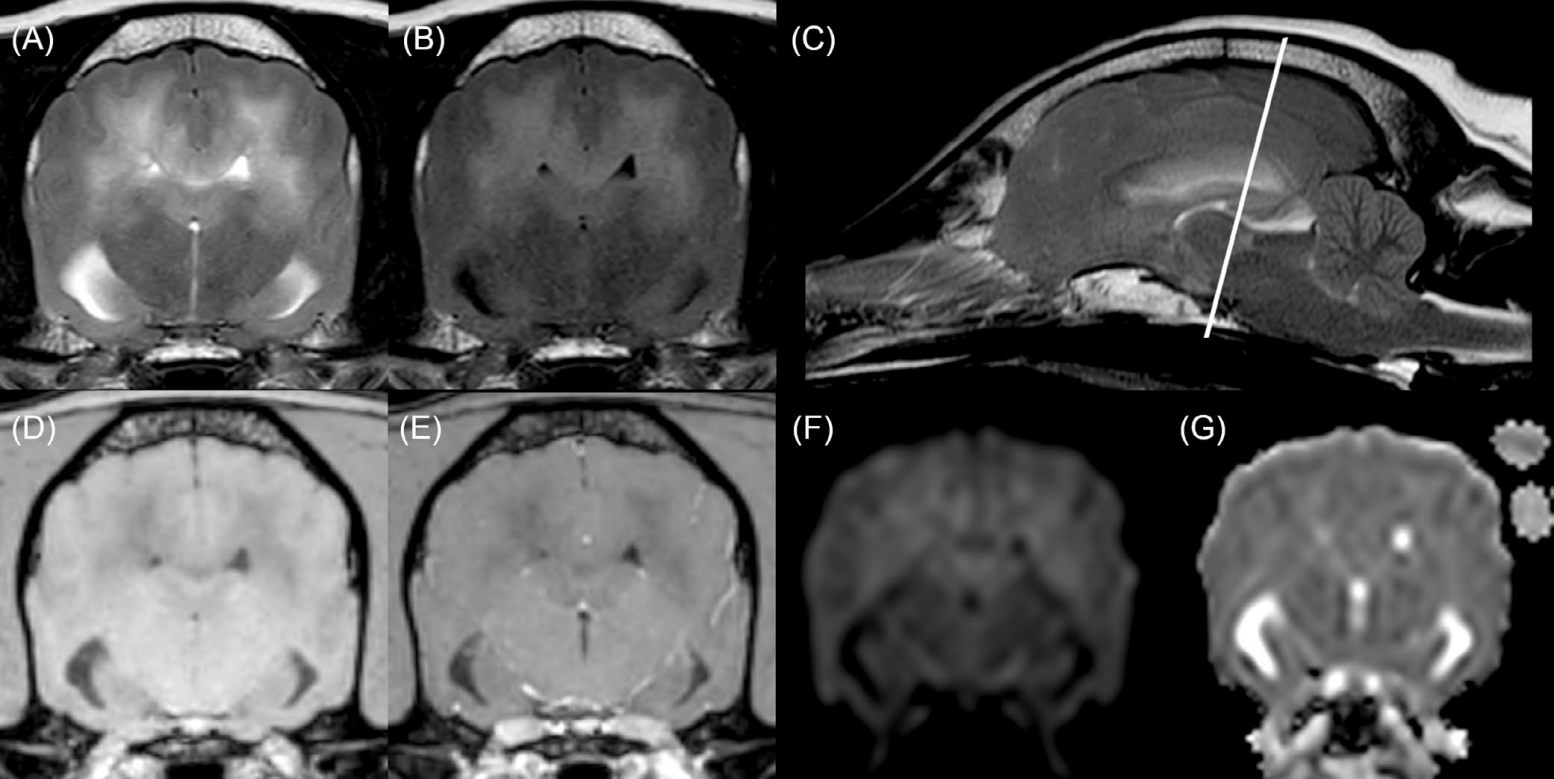
Transverse T2‐weighted (T2W); (A), fluid‐attenuated inversion recovery (FLAIR) (B), sagittal T2W (C) sequences, transverse T1‐weighted (T1W) sequence before (D) and after (E) contrast medium administration, diffusion‐weighted imaging (DWI) (D), and apparent diffusion coefficient (ADC) map (E). The white line in (C) indicates the location of the other transverse planes. Bilaterally, the white matter is diffusely T2W‐ (A, C), FLAIR‐ (B) hyperintense, and T1W‐ (D) iso‐ to hypointense, without contrast enhancement (E) of the lesion. No discrete mass is discernible. Flattened gyri and sulci are visible in the transverse views, caudal transtentorial and foramen magnum herniation in the sagittal plane. There is no evidence of a restrictive diffusion pattern.

The MRI scan revealed extensive, bilateral, ill‐defined T2W‐ and FLAIR‐hyperintensity of the white matter from the parietal up to the occipital lobe, following especially the dorsal parts including the corpus callosum and corona radiata. Cortical gray matter and meninges were spared. The described changes were T1W iso‐ to hypointense. There was no evidence of a restrictive diffusion pattern or abnormal contrast enhancement. The affected white matter was moderately increased in volume, with flattened overlying gyri and sulci. Caudal transtentorial and foramen magnum herniation were noted. There was no evidence of a discrete mass in any sequence (Figure 1).

The findings were summarized as an extensive, bilateral, symmetric encephalopathy focused on the white matter of the parietal and occipital lobes. The most likely causes included a metabolic‐toxic disorder (e.g., exposure to an unknown toxin), neoplasia (e.g., gliomatosis cerebri, round cell neoplasia), or inflammatory disease (e.g., meningoencephalitis of unknown origin [MUO]). Degenerative encephalopathy was considered unlikely due to the involvement of white matter in combination with a severe mass effect [16].

**FIGURE 2:**
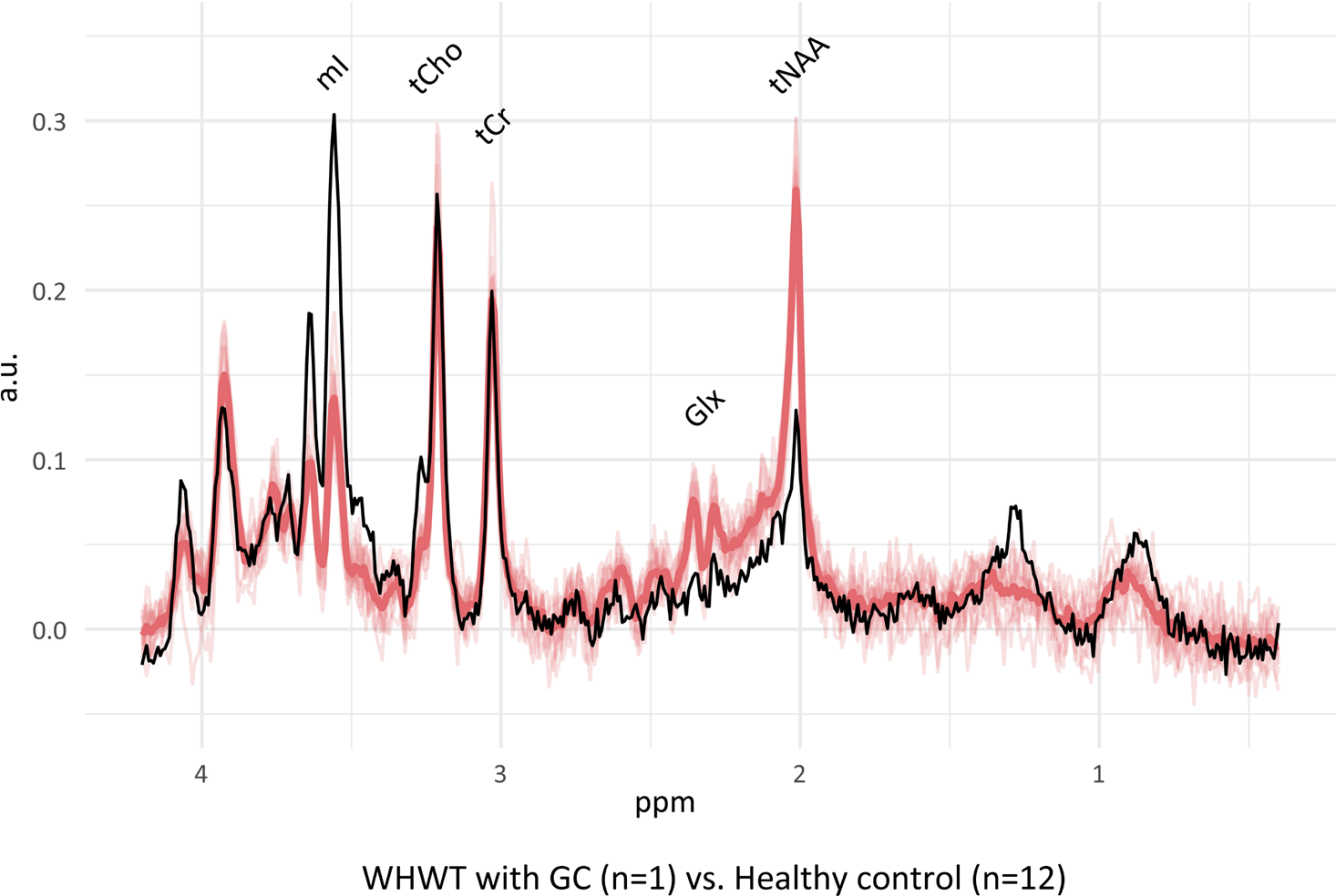
Comparison of the spectrum obtained from the WHWT with GC at the level of the left parietal area (black line) with the mean spectrum of the healthy control dogs at the level of the thalamus [28] (red line). All individual spectra from the healthy control dogs used to calculate the mean are displayed in light red. Compared to the controls, the WHWT with GC shows a markedly increased mI peak and a reduced tNAA peak. Relevant metabolite peaks are labeled in the figure. All spectra are scaled to the respective water signal for visualization purposes. GC, Gliomatosis cerebri; Glx, Glutamate‐glutamine complex; mI, Myo‐inositol; tCho, total choline; tCre, total creatine; tNAA, *N*‐acetyl‐aspartate containing compounds; WHWT, West Highland white terrier.

Single‐voxel point‐resolved spectroscopy (PRESS) was performed before contrast media administration at the level of the left parietal area with a voxel size of 1.8 cm^3^ (10 × 12 × 15 mm^3^) using a previously described optimized protocol [28]. Spectra were obtained using the following parameters: TE: 30.5 ms; repetition time: 2000 ms; number of signal averages (NSA): 256; and bandwidth: 2000 Hz. Metabolite concentrations were estimated as previously described using an automated spectral fitting algorithm (linear combination model, LCModel, version 6.3‐1L, Provencher, Oakville, ON, Canada) [29]. Metabolite concentration ratios were then calculated either with the water or the tCr as reference signal, exactly as described in a previous study [28]. The water signal was acquired interleaved with the metabolites signal from the same area using the same settings but with only 4 NSA. These water ratios give rough estimates of mmol/L concentrations. Spectra and values of the case were compared to the reference values of the thalamus of healthy control dogs measured with the same protocol [28]. All metabolite ratios outside of the range of the control dogs are shown in Tables S2 and S3 [28]. What stands out in particular are the markedly elevated mI levels (twice the maximum of the normative data) and the highly reduced tNAA. These changes are also clear in the visual comparison between the spectrum of the dog with GC and those of the healthy controls (Figure 2). A marked reduction in glutamate and glutamine (Glx), along with an increase in glucose (Glc) was also observed. When using tCr or water as reference signal, there are no deviations in the interpretation except for tCho. With regard to tCr, tCho lies within the normal range; relative to water, tCho is slightly above the normative values.

Cerebrospinal fluid (CSF) was collected via lumbar puncture and revealed a protein count of 28 g/dL (reference range < 40 g/dL) and mild mixed mononuclear pleocytosis with 10 nucleated cells (reference range < 5 cells/μL). The erythrocyte count was 419/μL and no atypical cells were detected. CSF PCR assay was negative for Tick‐borne encephalitis virus, Toxoplasma gondii, and Neospora caninum.

The initial treatment consisted of prednisolone (1 mg/kg q12h; Prednisolon Streuli, Streuli Pharma AG, Uznach, Switzerland) and fluids (3 mL/kg/h; PlasmaLyte, Baxter Healthcare Ltd., Thetford Norfolk, United Kingdom) administered intravenously. Despite the treatment, the dog's neurological condition progressively worsened, and the dog developed left‐sided pleurothotonus, circling to the left, vestibular ataxia, and severe proprioceptive deficits. Due to acute worsening despite treatment, an image‐guided brain biopsy of the left parietal lobe was performed under general anesthesia using an advanced neuronavigational system (StealthStation S8, Medtronic Schweiz AG, Münchenbuchsee, Switzerland) to obtain a histological diagnosis. The sampled tissue was submitted to the Division of Neurological Sciences of the University of Berne, Switzerland for histopathological analysis.

**FIGURE 3:**
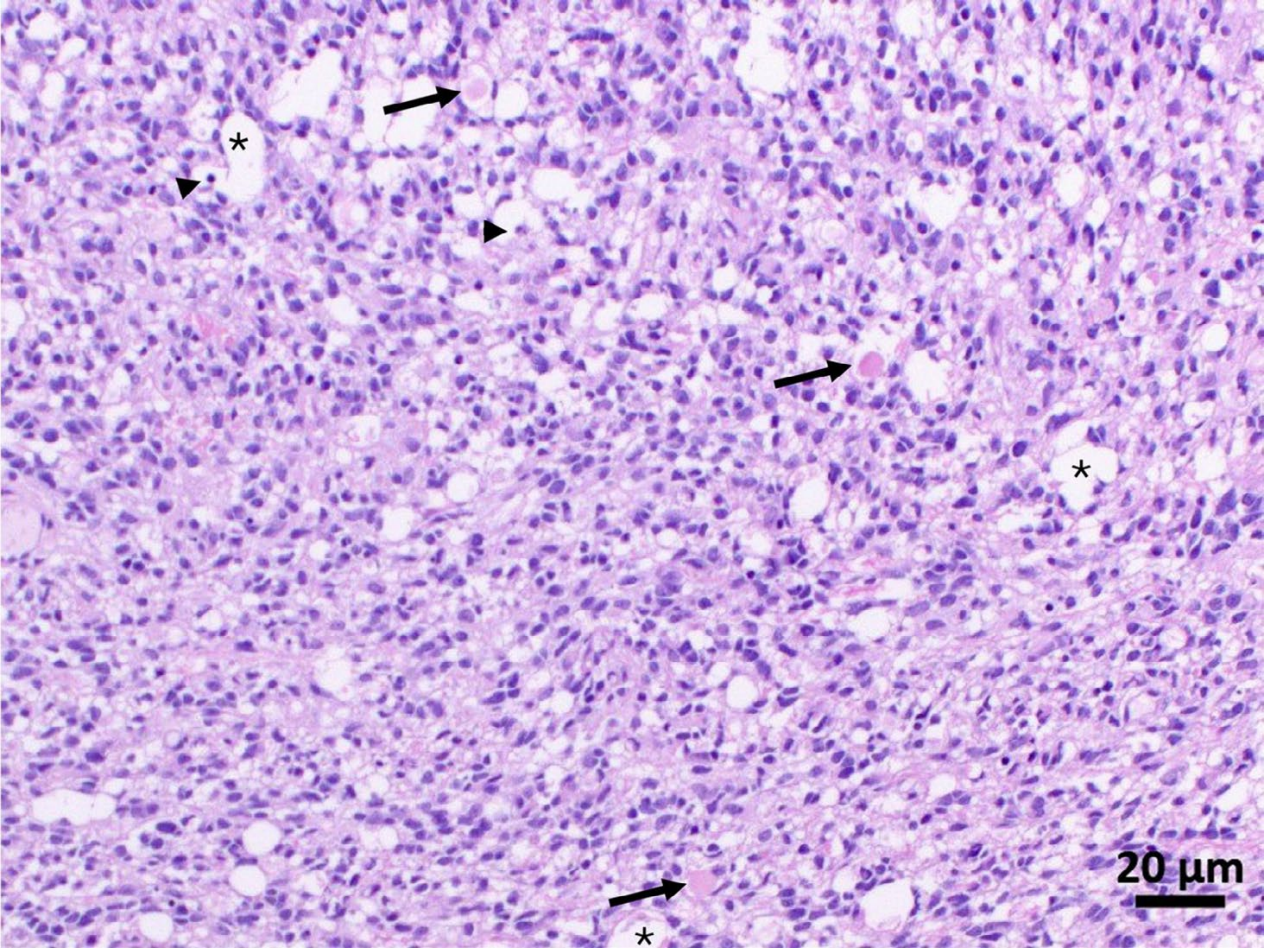
Hematoxylin and eosin‐stained section of the paraffin embedded biopsy. Histopathology reveals a dense population of neoplastic cells with hyperchromatic nuclei infiltrating the white matter. The lower half of the image shows the neoplastic cell arranged in bundles and streams, aligned along axonal pathways. Asterisks mark dilated myelin sheaths, arrows indicate axonal spheroids, and arrowheads highlight pyknotic nuclei (indicative of single cell necrosis). 200× magnification.

Macroscopically, all three submitted biopsy samples displayed a gray‐beige discoloration and a firm‐elastic consistency. One biopsy sample additionally exhibited a punctiform brownish discoloration. The biopsies were obtained from the boundary of cortex to subcortical white matter and predominantly comprised white matter with a smaller proportion of gray matter. Histologically, numerous elongated neoplastic cells were observed infiltrating primarily the white matter. These cells were frequently arranged in dense bundles and streams aligned along axonal tracts (Figure 3). The neoplastic cells had hyperchromatic, oval to cigar‐shaped nuclei, scant eosinophilic cytoplasm with long cellular processes, and poorly defined cell boundaries. The infiltrated white matter appeared pale and edematous, with the presence of multifocal dilated myelin sheaths containing either axonal spheroids or foamy macrophages. Multifocal karyopyknosis and karyorrhexis were evident, indicating single cell necrosis. Neoplastic glial cells also invaded the gray matter, forming multifocal perineuronal secondary structures of Scherer. The histopathological findings were consistent with a diagnosis of diffuse anaplastic astrocytoma, WHO grade III. Combined with the MRI findings, these results were indicative of a diffuse astrocytoma with a GC growth pattern.

Based on human medical literature [2, 30, 31, 32], chemoradiotherapy (temozolomide 150 mg/m^2^ and lomustine 70 mg/m^2^ in combination with a whole brain radiation protocol [10 × 3 Gy]) was recommended, and the owner opted to proceed. The dog died shortly after the first radiotherapy treatment session and before the first scheduled administration of chemotherapy. The owners declined postmortem histopathological examination.

## 3 Discussion

We describe the 1H‐MRS findings at 3 T of a dog with biopsy‐confirmed GC. In addition to the previously reported reduced tNAA levels, we observed markedly elevated mI levels compared to healthy control dogs. The marked increase in mI concentration in the MR spectra is reported in humans with GC but has not yet been described in dogs with this condition [24, 25, 26].

Due to the heterogeneous and widespread distribution of GC, the clinical presentation and conventional MRI findings can be nonspecific and mimic other diffuse and bilateral central nervous system diseases affecting the white matter or both white and gray matter. Similar MRI features have been described in dogs with MUO, hepatic or hypertensive encephalopathy, L‐2‐hydroxyglutaric aciduria, or ethylene glycol intoxication [16, 33]. Another possible differential in this case is bromethalin intoxication, in which diffuse, bilateral changes in the white matter with an increase in brain volume are reported [16]. Interestingly, in the present case, no restricted diffusion was observed based on the DWI and ADC map. This contrasts with reported cases of bromethalin intoxications, where restricted diffusion in the white matter has been detected [16].

Proton magnetic resonance spectroscopy is widely used in humans to achieve additional biochemical information for the specific region of interest, to increase the accuracy of diagnosis of several intracranial pathologies such as inflammatory, ischemic, neoplastic, or metabolic brain disorders, as well as to improve treatment monitoring, facilitate biopsy, radiotherapy, or surgical planning, and provide prognostic biomarkers [18, 19, 20, 34]. The number of metabolites that can be identified with 1H‐MRS depends on the investigated tissue, the chosen TE, and the available field strength [22, 23]. In veterinary medicine, the use of 1H‐MRS is limited, but its clinical importance is growing due to the increasing availability of high‐field magnets.

Comparing the 1H‐MRS findings of this GC case with those from the differential diagnoses considered based on morphological MRI reveals the following differences: in cases of MUO and tick‐borne encephalitis in dogs, 1H‐MRS findings also included reduced tNAA‐to‐creatine ratios [35, 36]; however, in contrast to our case, mI is either unchanged [35, 37] or decreased [36]. In dogs with hepatic encephalopathy, Glx levels are markedly increased and mI levels clearly reduced [38]. In reported cases of glial cell tumors and lymphoma, increased tCho, reduced tCr, and reduced tNAA have been described [36]. Severely increased mI, as observed in our case, has not been reported in the veterinary literature. 1H‐MRS findings have neither been reported in hypertensive encephalopathy nor in degenerative brain diseases with volume gain, such as L‐2‐hydroxyglutaric aciduria or ethylene glycol intoxication in dogs. In humans, increased mI together with an unusual singlet at 2.65 ppm has been described in a single case of L‐2‐hydroxyglutaric aciduria [39]. In hypertensive encephalopathy, mI concentrations have not been documented, and depending on the timing, tNAA might be reduced and tCho elevated [40]. No 1H‐MRS data are available for ethylene glycol intoxications; however, considering the dog was presented 3 weeks after the onset of the clinical signs, renal failure would have been expected if it were the cause [41].

Myo‐inositol is a glial marker, with increased levels linked to glial proliferation or an increase in glial cell size. Myo‐inositol peaks are elevated in gliosis and astrocytosis [22, 23, 42]. In human medicine, mI has been described as a useful complementary tool in grading gliomas and in the diagnosis of GC [25, 43], with higher levels typically seen in low‐grade rather than high‐grade astrocytoma. This is thought to be due to the lack of activation of phosphatidylinositol metabolism and mI accumulation at the level of the neoplasm [22]. Myo‐inositol is reported to be a useful marker for GC in humans. However, the significant increase in mI concentration in high‐grade astrocytoma with a GC growth pattern is not well understood and might suggest that the growth pattern may influence mI levels more than tumor grade [25].

*N*‐acetyl‐aspartate is considered a marker of neuronal and axonal integrity, and a decreased concentration is a sign of neuronal loss or degradation [21]. In the presented case, histology confirmed axonal and neuronal loss, which might explain the highly reduced tNAA levels.

Choline is a marker of membrane density and integrity. In malignant gliomas, tCho peaks are typically elevated due to increased cellularity. However, increased tCho concentrations and elevated tCho‐to‐creatine ratios have also been reported in dogs with non‐infectious meningoencephalitis and Lafora disease [36, 44]. Studies of GC in humans have described elevated mI levels with normal tCho peaks [25], a pattern that parallels the findings in the described dog with GC, where tCho was only mildly elevated and only when referenced to water.

Limitations of this case report include the lack of a postmortem whole brain histopathological examination. This could have confirmed the presence of astrocytoma in multiple brain areas. Nevertheless, the biopsies were consistent with a diffuse high‐grade astrocytoma in the region of the MRS voxel, and the described MRI features were highly suggestive of GC. The spectra obtained from the left parietal lobe were compared with the reference values of the thalamic region. However, in another study, the NAA and the mI levels did not differ significantly between the parietal and thalamic areas [29]. Finally, only one dog with GC was examined, which may not reflect the 1H‐MRS characteristics of the entire dog population suffering from this disease.

## 4 Conclusion

Highly elevated mI levels (relative to tCr or water) and decreased tNAA levels in 1H‐MRS might support the imaging‐based diagnosis of GC even when histopathological examination is unavailable. These findings could aid clinical decision‐making, biopsy planning, and treatment monitoring in dogs with GC.

## Disclosure

Authors declare no off‐label use of antimicrobials.

## Ethics Statement

Authors declare no institutional animal care and use committee or other approval was needed. Authors declare human ethics approval was not needed.

## Conflicts of Interest

The authors declare no conflicts of interest.

## Supporting information


**Table S1:** MRI protocol parameters. Acquired before and after contrast medium administration.
**Table S2:** Metabolite concentrations derived using water as the reference signal. Shown are only those metabolites for which the concentration in the dog with GC fell outside the range observed in healthy control dogs. Part A lists metabolites elevated relative to normative data; part B shows metabolites with reduced concentrations. Due to the spectral overlap between myo‐inositol (mI) and glycine (Gly), the values for their summed signal (mI+Gly) are also reported. Mean relative Cramér–Rao lower bounds (CRLBs) for each metabolite in healthy control dogs are provided, along with the corresponding relative CRLBs for the GC case. CRLBs indicate the minimum possible theoretical uncertainty based on model fit and noise; lower CRLB values reflect greater confidence in the estimated concentrations.
**Table S3:** Metabolite concentration ratios relative to total creatine (tCr). Shown are only those metabolites for which the ratio in the dog with GC fell outside the range observed in healthy control dogs. The resulting set of metabolites corresponds to those in Table S2, except for total choline (tCho), which was within the normative range when expressed relative to tCr. For comparison, tCho is still included in the table.
