# Supplementary material for: Single‐Voxel Proton Magnetic Resonance Spectroscopy Findings at 3 Tesla in a Dog With Gliomatosis Cerebri
Source: J Vet Intern Med. 2025 Sep 25;39(6):e70210. doi: 10.1111/jvim.70210 (PMC12462539; doi:10.1111/jvim.70210)
Supplement: Supplementary file 1 — Table S1: MRI protocol parameters. Acquired before and after contrast medium administration. Table S2: Metabolite concentrations derived using water as the reference signal. Shown are only those metabolites for which the concentration in the dog with GC fell outside the range observed in healthy control dogs. Part A lists metabolites elevated relative to normative data; part B shows metabolites with reduced concentrations. Due to the spectral overlap between myo‐inositol (mI) and glycine (Gly), the values for their summed signal (mI+Gly) are also reported. Mean relative Cramér–Rao lower bounds (CRLBs) for each metabolite in healthy control dogs are provided, along with the corresponding relative CRLBs for the GC case. CRLBs indicate the minimum possible theoretical uncertainty based on model fit and noise; lower CRLB values reflect greater confidence in the estimated concentrations. Table S3: Metabolite concentration ratios relative to total creatine (tCr). Shown are only those metabolites for which the ratio in the dog with GC fell outside the range observed in healthy control dogs. The resulting set of metabolites corresponds to those in Table S2, except for total choline (tCho), which was within the normative range when expressed relative to tCr. For comparison, tCho is still included in the table. [file JVIM-39-e70210-s001.docx]

**Supplementary Table S1 -** MRI protocol parameters. Acquired before and after contrast medium administration.

| **Location** | **Sequence** | **Plane** | **TE (ms)** | **TR (ms)** | **ST (mm)** |
| --- | --- | --- | --- | --- | --- |
| Brain | T2W-TSE | transverse | 100 | 4017 | 2.5 |
|  | T2W-TSE | sagittal | 100 | 2357 | 2.5 |
|  | T2W-TSE | dorsal | 100 | 2410 | 2.5 |
|  | T1W-TFE | 3D | 5.9 | 12.9 | 0.6 |
|  | FLAIR | transverse | 125 | 11000 | 2.5 |
|  | DWI^a^ | transverse | 75 | 4691-4699 | 2.0 |
|  | SWI | transverse | 7.2 | 31 | 2.0 |
|  | T1W-TFE +C | 3D | 5.9 | 12.9 | 0.6 |

Abbreviations: 3D, 3-dimensional; DWI, diffusion-weighted imaging; FLAIR, fluid-attenuated inversion recovery; ST, slice thickness; SWI, susceptibility-weighted imaging; TE, echo time; TR, repetition time; T1W-TFE (+C), T1-weighted turbo field echo (after contrast medium administration); T2W-TSE, T2-weighted turbo spin echo. ^a^B-values set at 0 and 1000 s/mm^2^.

**Supplementary Table S2 –** Metabolite concentrations derived using water as the reference signal. Shown are only those metabolites for which the concentration in the dog with GC fell outside the range observed in healthy control dogs. Part A lists metabolites elevated relative to normative data; part B shows metabolites with reduced concentrations. Due to the spectral overlap between myo-inositol (mI) and glycine (Gly), the values for their summed signal (mI+Gly) are also reported. Mean relative Cramér–Rao lower bounds (CRLBs) for each metabolite in healthy control dogs are provided, along with the corresponding relative CRLBs for the GC case. CRLBs indicate the minimum possible theoretical uncertainty based on model fit and noise; lower CRLB values reflect greater confidence in the estimated concentrations.

|  | **Healthy control (n=12)** | | | | | **WHWT with GC (n=1)** | |
| --- | --- | --- | --- | --- | --- | --- | --- |
| **Metabolite** | Mean | SD | Min | Max | Mean CRLB (%) | Value | CRLB (%) |
| **A** |  |  |  |  |  |  |  |
| mI+Gly | 8.49 | 0.81 | 7.26 | 9.54 | 4.75 | 21.56 | 2 |
| mI | 7.30 | 1.11 | 4.77 | 8.63 | 9.08 | 17.99 | 3 |
| tCho | 2.61 | 0.23 | 2.21 | 2.92 | 4.00 | 3.03 | 3 |
| Glc | 1.20 | 0.82 | 0.00 | 2.60 | 155.83 | 3.94 | 11 |
| Lip13 | 4.90 | 3.05 | 0.73 | 11.24 | 63.00 | 13.57 | 21 |
| **B** |  |  |  |  |  |  |  |
| tNAA | 8.66 | 0.54 | 8.00 | 9.71 | 4.42 | 3.40 | 9 |
| Glx | 12.79 | 0.93 | 11.45 | 14.50 | 7.58 | 5.68 | 15 |

Abbreviations: GC, Gliomatosis cerebri; Glc, Glucose; Glx, Glutamate-glutamine complex; Gly, Glycine; Lip13, signals from mobile macromolecules or mobile lipids at around 1.3 ppm; mI, Myo-inositol; tCho, Total choline; tNAA, *N*-acetyl-aspartate containing compunds; WHWT, West Highland white terrier.

**Supplementary Table S3 –** Metabolite concentration ratios relative to total creatine (tCr). Shown are only those metabolites for which the ratio in the dog with GC fell outside the range observed in healthy control dogs. The resulting set of metabolites corresponds to those in Table S2, except for total choline (tCho), which was within the normative range when expressed relative to tCr. For comparison, tCho is still included in the table.

|  | **Healthy control (n=12)** | | | | | **WHWT with GC (n=1)** | |
| --- | --- | --- | --- | --- | --- | --- | --- |
| **Metabolite** | Mean | SD | Min | Max | Mean  CRLB (%) | Value | CRLB (%) |
| **A** |  |  |  |  |  |  |  |
| mI+Gly | 1.18 | 0.11 | 0.92 | 1.34 | 4.75 | 3.03 | 2 |
| mI | 1.01 | 0.12 | 0.80 | 1.19 | 9.08 | 2.53 | 3 |
| tCho | 0.36 | 0.05 | 0.29 | 0.47 | 4.00 | 0.43 | 3 |
| Glc | 0.17 | 0.12 | 0.00 | 0.38 | 155.83 | 0.55 | 11 |
| Lip13 | 0.67 | 0.41 | 0.12 | 1.55 | 63.00 | 1.90 | 21 |
| **B** |  |  |  |  |  |  |  |
| tNAA | 1.21 | 0.17 | 0.97 | 1.62 | 4.42 | 0.48 | 8 |
| Glx | 1.78 | 0.22 | 1.56 | 2.32 | 7.58 | 0.80 | 15 |

Abbreviations: GC, Gliomatosis cerebri; Glc, Glucose; Glx, Glutamate-glutamine complex; Gly, Glycine; Lip13, signals from mobile macromolecules or mobile lipids at around 1.3 ppm; mI, Myo-inositol; tCr, Total creatine; tCho, Total choline; tNAA, *N*-acetyl-aspartate containing compounds; WHWT, West Highland white terrier.
